# Prevention of Malaria in Pregnant Women and Its Effects on Maternal and Child Health, the Case of Centre Hospitalier de Kingasani II in the Democratic Republic of the Congo

**DOI:** 10.3390/tropicalmed9050092

**Published:** 2024-04-23

**Authors:** Japhet Kabalu Tshiongo, Trésor Zola Matuvanga, Patrick Mitashi, Vivi Maketa, Henk D. F. H. Schallig, Petra F. Mens, Hypolite Muhindo Mavoko, Junior Matangila Rika

**Affiliations:** 1 Department of Tropical Medicine, University of Kinshasa (UNIKIN), Kinshasa 01306, Democratic Republic of the Congo; zola.matuvanga@unikin.ac.cd (T.Z.M.); patrick.mitashi@unikin.ac.cd (P.M.); vivi.maketa@unikin.ac.cd (V.M.); hypolite.muhindo@unikin.ac.cd (H.M.M.); junior.matangila@unikin.ac.cd (J.M.R.); 2 Laboratory for Experimental Parasitology, Department of Medical Microbiology and Infection Prevention, Amsterdam University Medical Centre, 1105 AZ Amsterdam, The Netherlands; h.d.schallig@amsterdamumc.nl (H.D.F.H.S.); p.f.mens@amsterdamumc.nl (P.F.M.); 3 Amsterdam Institute for Immunology and Infectious Diseases, 1105 AZ Amsterdam, The Netherlands

**Keywords:** intermittent preventive treatment, gestational malaria, insecticide-treated nets use, pregnant women, birth weight, Democratic Republic of the Congo

## Abstract

This study aimed to evaluate scientific evidence of the benefit of the use of insecticide-treated nets (ITNs) and Intermittent preventive treatment (IPT) on the birth weight of newborns and the hemoglobin level of the mother when used to prevent malaria during pregnancy. This cross-sectional analytical study was conducted on 467 hospitalized women in the Maternity Ward of Centre Hospitalier de Kingasani II, in the Democratic Republic of the Congo. Data were collected using a structured questionnaire that was pre-tested during a face-to-face interview. Apart from basic statistics, the chi-square test was used to compare proportions. Multivariate analysis (logistic regression) was used to identify variables significantly associated with the 95% confidence interval (CI). The ITN ownership rate was 81% (95% CI: 77–84) and the ITN use rate was 66% (95% CI: 62–70). Sixty-five percent (95% CI: 60–69) reported having received at least three doses of IPT during pregnancy with sulfadoxine-pyramethemine (IPTp-SP). There was a statistically significant difference in hemoglobin levels between hospitalized women who did not use the ITN (9.4 g/dL IIQ: 8.7–9.9) and those who did (11 g/dL IIQ: 9.8–12.2). The non-use of the ITN was associated with low birth weight (aOR = 3.6; 95% CI: 2.1–6.2; *p* < 0.001) and anemia in pregnant women (cOR = 2.41; 95% CI: 1.16–5.01; *p* = 0.018). The use of ITN and taking at least three doses of ITP during pregnancy are associated with good birth weight. The number of doses of IPTp received during antenatal care is associated with the maternal hemoglobin level in the third trimester of pregnancy.

## 1. Introduction

Malaria in pregnant women (MiP) is a major public health problem with significant health risks for mothers and their offspring. It is responsible for 20% of stillbirths, 11% of all neonatal deaths, and 10,000 maternal deaths in sub-Saharan Africa (SSA) among the 55 million women who become pregnant each year [1,2,3,4,5]. Each year, an estimated 25 million pregnancies in SSA are at risk for malaria, which can have deleterious effects on the mother and the fetus in terms of morbidity and mortality. Sequelae of MiP include anemia, stillbirth, and intrauterine growth restriction [6,7].

In 2018, malaria mortality was estimated at 435,000 deaths worldwide, and the Democratic Republic of the Congo (DRC) counted 18,030 deaths [2]. Despite various control strategies, the disease remains a serious health problem in DRC, which accounts for more than 35% of malaria deaths worldwide. Malaria is identified as one of the most important causes of anemia in pregnant women, whose prevalence is around 60% in Kinshasa [6,7].

Malaria has maternal, perinatal, and neonatal effects during pregnancy. Whether symptomatic or not, *P. falciparum* infections during pregnancy significantly increase the risk of anemia in the mother [8,9,10,11,12]. Severe anemia is more often seen in a high-prevalence malaria area and more in primigravida than in multigravida. Malaria infections acquired in the first or second trimester of pregnancy increase the risk of anemia [13]. During the perinatal period, MiP increases the risk of low birth weight [8,11,12]. Congenital malaria can occur in newborns and may contribute to infant morbidity [14]. This is confirmed by the reduction in neonatal mortality of up to 60% observed after the implementation of preventive interventions (i.e., intermittent preventive treatment during pregnancy with sulfadoxine-pyrimethamine (IPTp-SP), and the use of insecticide-treated nets (ITNs)) in pregnant women [14,15,16]. The DRC National Malaria Control Program (NMCP) advises IPT-SP from the second trimester of pregnancy, with at least four monthly doses of 1500 mg/75 mg SP until delivery [17].

In order to reduce the high burden of malaria during pregnancy, the World Health Organization (WHO) recommends intensifying the preventive strategy based on ITN ownership and use as well as the coverage of pregnant women with IPTp-SP under directly observed treatment during antenatal care (ANC), with the aim of capitalizing on pregnant women’s attendance at ANC [2,18,19].

The DRC has subscribed to the WHO Global Technical Strategy for Malaria Control 2016–2030. For its operationalization, since 2016, the country has developed and implemented a national strategic plan (NSP) 2016–2020 through the NMCP, aiming to reduce cases and deaths by 40% compared to the 2015 situation [6]. This strategic plan underwent a mid-term evaluation whose quantitative results did not show the expected level of performance.

However, preliminary reports from the 2017–2018 Multi Indicators Cluster Survey (MICS) showed that 52.4% of pregnant women slept under an ITN the night before the interview (i.e., 60.4% in urban areas and 47.2% in rural areas) [4]. This use rate was 74.8% in Kinshasa (urban). The percentage of pregnant women who took at least one dose of IPTp-SP during at least one ANC visit was 56% (79% in Kinshasa). The percentage of those who took at least three doses of IPTp-SP in Kinshasa was 19.4% [5,6]. The demographic and health survey (DHS) II revealed that 60.2% of pregnant women had slept under an ITN the night before the interview (including 73.3% in Kinshasa) and 14.2% had received two or more doses of IPTp-SP during at least one ANC visit [6,7]. From the above, there is evidence of a regression in the appropriation of ITN use and little progress in adherence to IPTp-SP by pregnant women compared to the years before 2014 and 2007 in the country, despite variations related to urban or rural scenarios.

Hence, it is important to reassess the level of ownership of the strategy and elucidate associated factors. Additionally, evaluating the effects of its utilization on the mother through hemoglobin levels and on the child through birth weight and APGAR (appearance, pulse, grimace, activity, and respiration) score. This study aims to evaluate scientific evidence of the benefit the use of this strategy has on birth weight, APGAR score, and hemoglobin level in the mother.

## 2. Materials and Methods

### 2.1. Study Area and Design

The study was conducted at Centre Hospitalier de Kingasani II (CHK) commonly known as the “Maternité de Soeurs” in the semi-rural district of Kinshasa. This health facility is located in the South-East of Kinshasa suburbs and has a high rate of pregnant women attendance and a high number of deliveries. This analytical cross-sectional study was conducted from 13 April to 20 June 2018

The women who gave birth at the CHK were enrolled until the sample size was reached. We kept 43% as the rate of ITN ownership by pregnant women, referring to a study conducted five years earlier by Matangila et al. on the same site, to calculate the sample size according to the formula used to calculate the minimum sample size in surveys [1,4]:

N = (Z^2^·P·Q)/d^2^

N = sample size; confidence interval at 95% (with Z = 1.96); P = percentage of ITN used among pregnant women; d = precision at 5%.

The sample size will be estimated by the formula:

Z = 1.96 (for 95% CI)

P = 43%

Q = 100% − 43% = 57%

d = precision = 5%.

N= ((1.96)^2^ × 43 × 57)/5^2^= 9314/25 = 373

To anticipate missing data and non-responses rate, we increased the sample size by 10%. Therefore, a minimal sample size of 410 admitted women was required for our study.

The following variables were collected by interviewing admitted women: age, parity, gravidity, number of ANC, SP dose, education level, occupation, marital status, ITN ownership, ITN importance, and use the night before the survey.

Medical records of the admitted pregnant women were reviewed to complete data collection for the following variables: occurrence of a febrile episode during pregnancy, birth weight, the mother’s last hemoglobin level before delivery, and APGAR (1st, 5th, and 10th min). APGAR scores were considered normal with values ≥ 7 and abnormal for lower values, i.e., <7.

### 2.2. Data Collection

The study team consisted of two physicians (including a supervisor) and two nurses. The survey targeted women in the immediate postpartum period, deemed to be in apparent good health who had delivered at CHK during the study period. A questionnaire was conducted by a trained team who spoke the local language.

### 2.3. Data Management and Analysis

Survey questionnaires were collected by the field supervisor and subsequently reviewed independently from data collectors to ensure completeness. A sequential form number was assigned to each record to link data from various hospitalized women. These questionnaires were then given to data-entry clerks to enter into the database using Excel. All personal information was removed before data entry. All data were entered independently by two independent data-entry clerks using a double-entry system and compared for accuracy. Subsequently, the dataset was validated for any inconsistencies or missing data, and final cleaning was performed. Data were entered and cleaned using Excel software (Microsoft Office 2016) and processed using SPSS version 20. Apart from basic statistics, Mann–Whitney and Chi-square tests were used for the comparison of means, medians, and proportions, respectively. A logistic regression model was used to identify independent variables associated with IPTp-SP use, ITN use, low birth weight, and maternal anemia.

### 2.4. Ethical Considerations

The current study obtained approval from the Public Health School Ethics Committee of the University of Kinshasa (approval number: ESP/CE 047/2017). Post-partum women received detailed information about the study’s objectives, and their informed consent was obtained through signed documents. For women not attending school, consent was signed via fingerprint, in presence of an impartial witness.

## 3. Results

### 3.1. General Characteristics of Respondents at CHK

A total of 467 admitted women participated in our survey at CHK. Their ages ranged from 15 to 43 years, with a median value of 24 years (IIQ: 20–32) (see Table 1). The study population was predominantly married women or women cohabiting with husbands (77.1%), and women under 25 years of age represented the largest age group (54%). Education levels were notably low, with 21% of women without or limited to primary school and only 27% having more than four ANC visits. There was a significant correlation between age and parity (r = 0.7; *p* < 0.001). Primiparous women were significantly younger (21.6 ± 4.5 years) than multiparous women (35.8 ± 4.7 years) (*p* < 0.001). Additionally, 42.6% of the women surveyed were housewives and had no professional occupation (see Table 1).

### 3.2. ITN Ownership, Use, and Associated Factors

Most admitted women reported receiving ITNs during mass distribution campaigns (64% (95% CI: 60–68)) or during ANC (31% (95% CI: 27–35)), while 5% (95% CI: 3–7) reported purchasing their ITN themselves. Having a secondary school education (aOR = 3.02, 95% CI: 1.71–5.37) increased the chance of having an ITN three-fold. Being employed was weakly associated with ITN ownership (see Table 2).

### 3.3. Rate of ITN Use by Respondents

The utilization rate of ITNs was 66% (95% CI: 62–70). Among women admitted for assessment, 75% (95% CI: 71–80) reported sharing a single ITN with two or more individuals during their recent pregnancy. Additionally, our survey revealed that nearly 45% (95% CI: 40–49) of admitted women reported leaving their ITNs at least three times during a single night.

In univariate analysis, factors significantly associated with ITN use included age > 25 years (cOR = 2.001; 95% CI: 1.31–3.05, *p* < 0.001), having attained a high school and university level of education (cOR = 3.35; 95% CI: 1.49–7.94, *p* = 0.004), and being married or cohabiting with a partner (cOR = 2.03; 95% CI: 1.23–3.33, *p* = 0.005) (see Table 3). However, after adjusting for potential confounders in the final model, only age > 25 years (aOR = 1.66; 95% CI: 1.03–2.68, *p* = 0.034) and attainment of education limited to high school and university (aOR = 2.81; 95% CI: 1.23–6.75, *p* = 0.01) remained significantly associated with ITN use (see Table 3).

### 3.4. IPTp-SP Use and Associated Factors

Among the admitted women surveyed, 92% reported receiving at least one dose of SP during ANC of the current pregnancy. A significant correlation was observed between the number of SP doses received and the number of ANC visits (r = 0.492; *p* < 0.001). The study found that 65% (95% CI: 60.4–69.4) received at least three doses; 20% (95% CI: 16.3–23.5) had received four doses; 22% (95% CI: 16.3–23.5) received two doses; and 13% (95% CI: 9.8–15.9) received a single dose. The number of admitted women who received at least three doses of SP (IPTp-3) increased with the number of ANCs attended (*p* < 0.001). In other words, women who attended at least four ANCs were significantly more likely to receive at least three doses of IPT-SP, (OR = 5.837; *p* < 0.001), compared with those who attended ANC less than four times.

Women who started ANC in the first trimester had received the recommended number of at least three doses of SP, whereas most of those who started ANC in the third trimester (45%) had received only one dose of SP. Specifically, the likelihood of receiving at least three doses of SP was three times higher in women who initiated ANC in the first trimester compared to those who initiated in the third trimester (OR = 3.09; *p* = 0.032). In univariate analysis, factors such as gravidity (cOR = 2.3; 95% CI: 1.98–2.68; *p* < 0.001), number of ANCs attended (cOR = 2.12; 95% CI: 1.8–2.39; *p* < 0.001), and ownership of ITNs (cOR = 7.94; 95% CI: 2.71–60.9); *p* = 0.04) were associated with the use of IPTp. However, in the final model, the number of ANCs (aOR = 1.47; 95% CI: 1.22–1.78; *p* < 0.001) remained significantly associated with IPTp-SP use (see Table 4).

### 3.5. Malaria Prevention Strategy and Impact on Birth Weight, Hemoglobin Level, and APGAR

Of all births, the proportion of low birth weight was 31.4% (95% CI: 26–34). The median birth weights were 1.9 kg, 2.52 kg, 2.80 kg, 2.90 kg, and 3.20 kg, respectively, for admitted women who reported taking 0, 1, 2, 3, and 4 doses of IPTp-SP. The difference was statistically significant *p* < 0.001. The median birth weight of newborns whose mothers reported using an ITN during pregnancy was 3.1 kg, while for those whose mothers did not use an ITN, it was 2.8 kg. The median birth was significantly higher (*p* < 0.001) in the group using ITNs.

In the univariate analysis, several factors demonstrated associations with low birth weight: age ≤ 25 years (cOR = 1.48; 95% CI:1.15–1.91, *p* = 0.002), not owning the ITN (cOR = 0.46; 95% CI: 0.3–0.72, *p* < 0.001), having at least one febrile episode during pregnancy (cOR = 2.43; 95% CI: 1.79–3.31, *p* < 0.001), unemployment (cOR = 1.66; 95% CI: 1.24–2.21, *p* = 0.003), living without a husband (cOR = 3.2; 95% CI: 1.24–2.21, *p* < 0.001), not using an ITN (cOR = 1.72; 95% CI: 1.22–2.4, *p* = 0.002), having at least one febrile episode during pregnancy (cOR = 2.43; 95% CI: 1.79–3.31, *p* = 0.002), parity (cOR = 1.31; 95% CI: 1.2–1.4, *p* < 0.001), and the number of IPT doses (cOR = 2.3; 95% CI: 1.92–2.91, *p* = 0.003). However, in the final model, only age (aOR = 0.52; 95% CI: 0.32–0.83, *p* = 0.006), not using an ITN (aOR = 3.6; 95% CI: 2.1–6.2, *p* = 0.004), and living without a husband (aOR = 3.65; 95% CI: 2.29–5.8, *p* < 0.001) remained significantly associated with low birth weight (see Table 5).

Only 162 had reported a hemoglobin level and almost one-third of them (36.42%, 95% CI: 29.01–43.83) had a hemoglobin value of less than 10 g/dL. The median hemoglobin levels according to the use of SP were 8.4 g/dL; 11g/dL, 10.30 g/dL, and 11.4 g/dL, respectively, for admitted women who reported taking 1, 2, 3, and 4 doses of IPTp-SP. The difference was not statistically significant (*p* = 0.47; 0.22 and 0.15).

Non-use of an ITN was associated with maternal anemia in the third trimester of pregnancy (cOR = 2.41; 95% CI: 1.16–5.01, *p* = 0.018) as was the number of ANC sessions attended by pregnant women (cOR = aOR:, *p* = 0.04) in the univariate analysis as well as in the final model (see Table 6).

### 3.6. Factors Associated with Anemia during Pregnancy

The median hemoglobin level (9.4 g/dL IIQ: 8.7–9.9) in respondents who did not use the ITN was lower than that of mothers who did use it (11 g/dL IIQ: 9.8–12.2) with a statistically significant difference (*p* = 0.026).

The number of febrile episodes appeared to decrease with IPT doses. Indeed, respondents who took at least four doses of IPTp-SP had fewer febrile episodes (median = 2) than those who took less than four doses. However, the observed difference was not statistically significant (*p* = 0.07). The median number of febrile episodes among respondents who used an ITN was lower (2) than among respondents who reported not using an ITN during pregnancy (3). This difference was not statistically significant (*p* = 0.07).

The majority of newborns had an APGAR score greater than 7/10 in the 1st (70.45% 95% CI: 66–75), 5th (60% 95% CI: 56–65), and 10th minutes (79% 95% CI: 74–84) (see Table 7).

We calculated the median APGAR scores in the first minute based on the number of times the respondents took SP during pregnancy. A median of 7/10 was found for those who took four doses versus 6.5/10 for those who reported taking only one dose, although this was not statistically significant.

The median APGAR score in the 5th min was 8/10 for those who had taken four doses versus 10/10 for those who reported not having taken SP. This difference was, however, not statistically significant (*p* = 0.19). The median APGAR score of newborns whose mothers reported taking no dose of SP (10/10) was higher than that of newborns whose mothers reported taking more than one dose (8/10; 9/10) but this difference was not statistically significant (*p* = 0.43). The median APGAR values in the 1st minute for pregnant women who had or had not used the ITN during pregnancy were the same (7/10) *p* = 0.05.

The median APGAR scores in the 5th minute for pregnant women who had or had not used the ITN during pregnancy were the same (8/10) *p* = 0.06. The median APGAR scores in the 10th minute for admitted women with and without ITN use during pregnancy were the same (8/10) *p* = 0.34.

## 4. Discussion

This study assessed the different determinants of the use of the malaria prevention strategy among pregnant women at CHK and the impact of this use on the health of the pregnant woman through hemoglobin levels in the third trimester as well as on the health of the unborn through birth weight and the APGAR score.

The study showed that 81% of pregnant women reported owning an ITN, demonstrating that the target had been reached for the rate of ITN ownership by pregnant women according to the national policy (NPS 2016–2020) [6].

However, the ITN ownership rate reported in this survey appears to be far higher than the ITN ownership rate for pregnant women in urban areas in the country (DRC) but is not far from reflecting the ITN ownership rate for pregnant women in the city of Kinshasa according to the 2017 and 2018 MICS surveys [4,5]. This rate is also higher than that reported by Matangila et al., 2013 in the same health facility [1]. This suggests that the mass distribution campaigns from 2013 to 2017 had a significant impact on ITN ownership among pregnant women. The most reported method of ownership was mass distribution at 64%, followed by routine distribution during ANCs. However, NCMP, in collaboration with the reproductive health program, advocates the routine distribution of ITNs to pregnant women during ANCs. This approach aims to establish ANCs as the preferred channel for women surveyed to acquire ITNs. This may suggest a low level of routine ITN distribution, which is likely related to ITN embezzlement at some distribution sites. In fact, ITN losses usually occur during transport and storage at the lower level, as ITNs are not categorized as drugs or diagnostic tests.

The ITN use rate, expressed by the proportion of pregnant women who slept under an ITN the night before admission for delivery, was 66%. A similar rate was found by Taremwa et al. 2017 in Uganda [20]. However, this finding is lower than that of the 2017 MICS survey in Kinshasa, which had shown 74% use among pregnant women. This difference can be explained by the fact that our survey was limited to women who had given birth and received ANC at the CHK health facility, whereas the MICS survey was a community-based study that took into account pregnant women who were or were not attending ANCs.

For education level limited to secondary school and mosquito ITN use by respondents, Pettifor et al., 2008 in Kinshasa (DRC), and Manirakiza et al., 2011 found the same association with ITN use by pregnant women [21,22]. The association may be due to the fact that educated mothers can easily read and understand information about malaria and ITN. In addition, educated mothers may refer to news reports through the media.

A comparison of DHS-DRC II results (2014) with the results of this study revealed a substantial increase in IPT-SP coverage from 33% to 92%, and from 15% to 64% for at least three doses. The proportion of women receiving three doses of IPT-SP rose in correlation with the number of ANCs attended. Women who had four or more ANCs were 5 times more likely to receive at least three doses of IPT-SP compared with those who had fewer ANCs. These observations corroborate the results reported by studies in Benin and Cameroon [23,24]. Early initiation of ANC in the first trimester was associated with a threefold increase in the likelihood of receiving three doses of IPT-SP, highlighting the importance of early ANC initiation for preventive care.

The proportion of low-birth-weight infants was 31.47%. This value is almost twice higher than that found in Kamina (14.3%) in Haut-Lomami Province by Kangulu et al., 2017 but close to that found by Kyamusugulwa et al., 2006 (27%) in Kama in Maniema [21,25]. The proportion of underweight newborns described in our study was 2 times higher than the WHO standard [26]. Non-use of ITNs during pregnancy and having experienced at least one febrile episode during pregnancy were strongly associated with low birth weight.

The prevalence of anemia was 36.42%. This value closely resembles the findings of Taylor et al. in pregnant women in DRC (32.3%), but is notably lower than the rates reported by Matangila et al. in the same healthcare facility (61.1%) and those reported in Cameroon by the WHO (49%) [1,27,28]. This can be explained by the fact that this study showed a progressive appropriation of the preventive strategy against malaria by pregnant women attending CHK for ANC, given that these studies cited are prior to the present study. Indeed, we found in this study that at least 92% of pregnant women had received at least one dose of SP during ANC, with a utilization rate of 66%. We found that being in ANC 1 to 3 times had a beneficial effect on maternal third-trimester hemoglobin levels. In several other studies, a decreased risk of anemia in mothers with at least four ANCs has been shown [8,18,19].

The majority of newborns had an APGAR score greater than 7 in the 5th minute. Mbanzulu and Kapepela reported normal APGAR scores in neonates infected with malaria parasites [29]. This is explained by the fact that despite the possibility of in utero malaria infection, infants are usually protected by specific antibodies transmitted from the mother.

The median APGAR scores in the 5th min versus the number of doses of SP taken by respondents were 8/10 for those who took four doses versus 10/10 for those who reported not taking SP. The difference was not statistically significant. The same was true for ITN use and the APGAR value in the fifth minute. Yet in some studies, malaria was significantly associated with the fifth-minute APGAR score [30,31]. Indeed, there may be an accumulation of parasitized red blood cells and subsequent inflammatory phenomena that may lead to the disruption of fetal-placental blood flow resulting in fetal hypoxia causing intrauterine growth restriction and birth asphyxia [30].

This study has limitations, including the lack of specific dates of SP administration, thereby hindering the ability to verify adherence to WHO-recommended dosing schedules during pregnancy. Additionally, the recruitment of admitted women from a single hospital may introduce selection bias, and the situation of pregnant women who did not attend CHK for ANC is unknown. However, this potential bias may be mitigated by the shared cultural characteristics and socioeconomic status among pregnant women or the population of Kingasani, as indicated by DHS 2014 data. Additionally, factors such as malaria, nutrient deficiencies, chronic inflammation, HIV, and soil-transmitted helminth infestation can also lead to anemia and low birth weight in pregnant women. However, these factors were not considered in the current study.

Despite these limitations, the study has the merit of being, to our knowledge, one of the few to have elucidated the operationalization of the national strategy for malaria prevention in pregnant women. The present study provides updated data on the level of utilization of ITNs, IPTp-SP, associated factors, and their impact on hemoglobin levels and low birth weight. This information can be used for the evaluation and implementation of IPTp-SP and guide policy decisions in DRC and other low-income countries.

## 5. Conclusions

Maternal hemoglobin levels in the third trimester of pregnancy are positively associated with the number of IPTp doses administered during ANC. The findings also reveal that the use of ITNs and the administration of at least three doses of IPT are linked to a healthy birth weight. This study emphasizes the need to enhance strategies to increase IPTp-SP coverage and the use of ITN among pregnant women in the DRC, identifying a crucial area for health interventions. Upcoming research should investigate additional approaches to enhance the update and impact of malaria prevention, thus contributing to global efforts to reduce the burden of malaria during pregnancy and its adverse outcomes.

## Figures and Tables

**Table 1:** Demographic and obstetric profile of enrolled women.

Variables	n	Percentage	CI 95%
**Age**			
≤25 years	253	54.2	50–59
>25 years	214	45.8	40–49
**Gravidity**			
1	181	38.8	35–43
2	97	20.8	17–25
≥3	188	40.4	36–45
**Parity**			
1	217	46.6	42–51
2	108	23.2	19–27
3	54	11.6	9–14
4	37	7.9	5–10
≥5	50	10.7	8–14
**Levels of education**			
No formal education and primary school	98	21	17–25
Secondary	315	67.5	29–38
High and university	54	11.6	9–15
**Occupation**			
Unemployed	199	42.6	38–47
With employment	268	57.4	53–62
**Matrimonial status**			
Single	105	22.9	19–27
Married or cohabiting with a partner	362	77.1	73–81
**Number of ANC**			
0	13	3	1–4
1	27	6	1–4
2	76	18	15–22
3	198	46	43–52
≥4	116	27	24–32
**Number of persons sleeping under ITN**			
1	229	69.8	60–68
2	69	21	12–18
3	30	9.2	2–11

ANC: Antenatal care; ITN: insecticide-treated nets; CI: confidence interval.

**Table 2:** Factors associated with ITN ownership among enrolled women.

Variable	n	Percentage	cOR	CI 95%	*p*	aOR	CI 95%	*p*
**Age**								
>25 years	214	87.9	2.84	1.75–4.71	<0.001	2.26	1.17–4.50	0.016
≤25 years	253	74.3	1					
**Gravidity**	466	80.5	1.18	1.04–1.37	0.01	1.14	0.80–1.77	0.48
**Levels of education**								
High and university	54	98.1	32.14	6.60–580.42	<0.001	16.03	3.11–294.28	0.008
Secondary	315	83.2	2.99	1.80–4.96	0.001	3.02	1.71–5.37	<0.001
No formal education and primary school	98	62.2	1					
**Matrimonal status**								
Married or cohabiting with a partner	291	84.5	2.25	1.35–3.69	0.001	1.81	1.00–3.24	0.04
**Single**	176	73.9	1					
**Occupation**								
With employment	268	85.8	2.19	1.38–3.51	0.001	1.53	0.91–2.60	0.10
Unemployed	199	73.4	1	0				
**Number of ANC**	430	80.5	1.43	1.15–1.77	<0.001	1.29	1.02–1.64	0.03
**Parity**	466	80.5	1.19	1.02–1.41	0.03	0.84	0.53–1.26	0.44

ITN: Insecticide-Treated Nets; ANC: Antenatal care; cOR: Crude Odds Ratio; aOR: Adjusted Odds Ratio; CI: Confidence Interval.

**Table 3:** Factors associated with ITN use.

Variable	n	Percentage	cOR	CI 95%	*p*	aOR	CI 95%	*p*
**Age**								
>25 years	186	73.1	2.001	1.31–3.05	<0.001	1.66	1.03–2.68	0.034
≤25 years	199	54.3	1					
**Gravidity**	242	63.2	1.002	0.91–1.10	0.96			
**Levels of education**								
Secondary	270	62.6	1.47	0.83–2.56	0.17	1.61	0.91–2.86	0.09
High and University	53	79.2	3.35	1.49–7.94	0.004	2.81	1.23–6.75	0.01
**No formal education and primary school**	62	53.2	1					
**Number of person sleeping under ITN**	325	63.5	0.91	0.64–1.3	0.63			
**Matrimonal status**								
Married or cohabiting with a partner	303	67.0	2.03	1.23–3.33	0.005	1.58	0.92–2.7	0.09
Single	82	50.0	1					
**Occupation**								
With employment	234	65.4	1.24	0.81–1.90	0.30			
Unemployed	151	60.3	1					
**Number of ANC**	354	64.7	1.26	0.81–1.97	0.43			
**Parity**	384	63.3	0.95	0.85–1.08	0.49			

ITN: Insecticide-Treated Nets; ANC: Antenatal care; cOR: Crude Odds Ratio; aOR: Adjusted Odds Ratio; CI: Confidence Interval.

**Table 4:** Factors associated with the use of IPTp-SP by women attendants during ANC.

Variable	n	Percentage	cOR	CI 95%	*p*	aOR	CI 95%	*p*
**Age**	461	92	3.63	1.97–1.20 × 10^1^	0.44			
**Gravidity**	458	91.1	2.3	1.98–2.68	<0.001	3.78	1.59–54.1	0.93
**Levels of education**	461	92		1.54–9.71	1			
**Matrimonal status**								
Single	103	92.2	1.04	0.46–2.3	0.91			
Married or living with a partener	358	91.9	1					
**Occupation**								
Unemployed	197	90.9	0.77	0.39–1.5	0.44			
With employment	264	92.8	1					
**Number of ANC**	423	91.5	2.12	1.8–2.39	<0.001	1.47	1.22–1.78	<0.001
**ITN Ownership**								
Yes	371	93.3	7.94	2.71–6.09 × 10^1^	0.04	3.14	1.05–1.5935	0.91
No	90	86.8	1					
**ITN use**								
Yes	242	93.4	4.25	1.94–2.2 × 10^1^	0.34			
No	138	92.8	1					
**Parity**	460	92	1.01	0.83–1.23	0.85			

IPTp-SP: Intermittent Preventive Treatment during pregnancy with Sulphadoxine-pyrimethamine; ITN: Insecticide-Treated Nets; ANC: Antenatal care; cOR: Crude Odds Ratio; aOR: Adjusted Odds Ratio; CI: Confidence Interval.

**Table 5:** Factors associated with low birth weight (<2500 g).

Variables	n	Percentage	cOR	CI 95%	*p*	aOR	CI 95%	*p*
**Age**								
≤25 years	251	40.2	1.48	1.15–1.91	0.002	0.52	0.32–0.83	0.006
>25 years	214	18.2	1					
**Gravidity**								
**Primigravid**	180	36.7	1.72	1.27–2.33	0.001			
**Multigravid**	284	25.7	1					
**Levels of education**								
No formal education and primary school	97	48.5	1.06	0.71–1.58	0.76			
Secondary	315	28.6	2.5	1.95–3.19	0.001			
High and university	53	56	1					
**ITN use**								
No	139	36.7	1.72	1.22–2.4	0.002	3.6	2.1–6.2	<0.001
Yes	244	13.1	1					
**Matrimonial status**								
Single	104	53.8	3.2	2.5–4.2	<0.001	3.65	2.29–5.8	<0.001
Married or cohabiting with partner	361	23.3	1					
**Occupation**								
Unemployed	197	37.6	1.66	1.24–2.21	<0.001			
With employment	268	24.7	1					
**Number of ANC**	428	70.6	1.2	1.2–1.37	<0.001			
**ITN ownership**								
No	91	68.1	0.46	0.3–0.72	<0.003			
Yes	374	20.9	1					
**Presence of at least one febrile episode during pregnancy**								
Yes	134	75.4	2.43	1.79–3.31	0.002			
No	329	11.9	1					
**Parity**	464	68.9	1.31	1.2–1.4	<0.001			
**IPTp-SP number**								
≤2	60	40.5	2.3	1.92–2.91	0.002			
≥3	59	22.1	1					

IPTp-SP: Intermittent Preventive Treatment during pregnancy with Sulphadoxine-pyrimethamine; ITN: Insecticide-Treated Nets; ANC: Antenatal Care; cOR: Crude Odds Ratio; aOR: Adjusted Odds Ratio; CI: Confidence Interval.

**Table 6:** Factors associated with anemia during pregnancy.

Variables	n	Percentage	cOR	CI 95%	*p*	aOR	CI 95%	*p*
**Age**	142	63.1	1.07	1.01–1.12	0.01	1.06	1.01–1.13	0.01
**Gravidity**	160	63.1	1.03	0.90–1.18	0.62			
**Abortion**								
No	119	36.1	0.91	0.44–1.9	0.82			
Yes	42	38.1	1					
**Levels of education**								
No Education	34	41.2	1.29	0.59–2.7	0.51			
With education	128	32.2	1					
**Insecticide use**								
No	92	34.8	0.84	0.44–1.61	0.62			
Yes	70	38.6	1					
**Matrimonial status**								
Single	30	50	2	0.89–4.4	0.09			
With partner	132	33.3	1					
**Occupation**								
No occupation	75	33.3	0.77	0.40–1.48	0.44			
With occupation	87	39.1	1					
**Number of ANC**								
1 to 3	111	37.8	0.6	0.41–0.89	0.01	0.45	0.21–0.97	0.04
≥4	38	31.5	1					
**ITN ownership**								
No	33	27.3	0.59	0.25–1.37	0.22			
Yes	129	38.8	1					
**IPTp**								
No	15	53.3	2.15	0.73–6.27	0.16			
Yes	147	34.7	1					
**Use of ITN**								
No	49	51.0	2.41	1.16–5.01	0.018			
Yes	83	30.1	1					
**Deworming medication**								
**No**	9	33.3	0.84	0.20–3.5	0.82			
**Yes**	151	37.1	1					
**Number of febrile episodes**								
≤1	43	41.9	1.37	0.67–2.79	0.38			
>2	119	34.5	1					
**Parity**	161	63.4	1.01	0.85–1.20	1.01			

ITN: Insecticide-Treated Nets; ANC: Antenatal care; cOR: Crude Odds Ratio; aOR: Adjusted Odds Ratio; CI: Confidence Interval.

**Table 7:** Proportion of newborns based on APGAR score in 1st, 5th, and 10th minutes.

APGAR 1st min	n	% (CI 95%)	APGAR 5th min	n	%(CI 95%)	APGAR 10th min	n	% (CI 95%)
<7	138	29.6 (25–34)	<7	186	39.82 (35–44)	<7	98	21.0 (16–26)
≥7	329	70.5(66–75)	≥7	281	60.18 (56–65)	≥7	369	79.0 (74–84)
Total	467	100		467	100		467	100

## Data Availability

Data are available upon reasonable request from the corresponding author.
